# Lancette Canadienne

**Published:** 1847-02

**Authors:** 


ARTICLE XVI.

"LANCETTE CANADIENNE."

We have received the first number of the "Lancette Canadienne," a native journal, published at Montreal, L. C., in the French language, and conducted by J. L. Leprohon, M.D. It is issued every two weeks, at $4 per annum, in a newspaper form.

The editor, in a modest and well written prospectus, sets forth the wants of a large number of physicians of Lower Canada who are of French origin, and have but an imperfect knowledge of the English language. There are also some in the United States, who for various reasons would, we are sure, be pleased to see a medical publication in the French language, and gain an acquaintance with the practice of the numerous skillful physicians of Canada. To such the "Lancette" will in every respect commend itself, its object and spirit being excellent, and the selections judicious and valuable. We wish it success.

